# Knowledge, attitudes and perceptions of a patient population on the COVID-19 vaccine rollout

**DOI:** 10.4102/hsag.v27i0.1845

**Published:** 2022-12-05

**Authors:** Makaira Purasram, Varsha Bangalee, Frasia Oosthuizen, Rajatheran Moodley

**Affiliations:** 1 Discipline of Pharmaceutical Sciences, Faculty of Health Sciences, University of KwaZulu-Natal, Durban, South Africa

**Keywords:** knowledge, attitudes, perceptions, hesitancies, COVID-19, Merebank, Wentworth and Bluff

## Abstract

**Background:**

The coronavirus disease 2019 (COVID-19) pandemic has had dire effects on South Africa. Vaccines against severe acute respiratory syndrome coronavirus 2 (SARS-CoV-2) are critical in the fight against COVID-19. This study is necessary to optimise vaccine acceptance.

**Aim:**

To determine the knowledge, attitudes and perceptions of a patient population in South Africa on the COVID-19 vaccine rollout.

**Setting:**

This study was conducted via a retail pharmacy in Merebank, Wentworth and Bluff (Ward 68), which is in the eThekwini Metropolitan Municipality in the KwaZulu-Natal province.

**Methods:**

A quantitative study was conducted using an online self-administered questionnaire between April 2021 to September 2021. There were a total of 430 participants. Data were collected on Google Forms, recorded in Microsoft Excel and analysed using descriptive and inferential statistics.

**Results:**

Knowledge of COVID-19 in the population was 81.86%. A total of 65% of participants stated that they would definitely take the COVID-19 vaccine, and 33.7% stated that they were hesitant to receive the vaccine. Reasons for hesitancies included concerns surrounding side effects of the vaccines, its safety and efficacy and the fast-tracking of the vaccine.

**Conclusion:**

Education campaigns need to be customised to provide the population with reliable and vetted vaccine information and address specific concerns or hesitancies present. Health care workers and the government need to work with religious leaders to improve public trust and confidence in the vaccine. To reach herd immunity and prevent increased morbidity rates, there needs to be a rise in vaccine acceptance across South Africa and globally.

**Contribution:**

With the intention of ensuring a successful COVID-19 vaccine rollout strategy in South Africa, it is of great importance to address the reasons for vaccine hesitancy and to determine the knowledge, attitudes and perceptions of the population on the COVID-19 vaccines. This study will therefore aid in developing strategies aimed at improving vaccine education and awareness, thereby resulting in a greater uptake of the COVID-19 vaccine by the population.

## Introduction

The coronavirus disease 2019 (COVID-19) pandemic has brought the world to a standstill. A vaccine against severe acute respiratory syndrome coronavirus 2 (SARS-CoV-2) is the best way to curtail this pandemic. It is for this reason that researchers have been working tirelessly to fast-track a safe and effective vaccine. The incredible success that researchers have had in getting vaccines to health care workers and patients at an unprecedented speed is a testament to modern medicine and technology. However, of equal importance is ensuring that these COVID-19 vaccines are equitably distributed through the global population so that herd immunity may be achieved. Herd immunity or herd protection occurs when a large portion of the population becomes immune to a virus, making it challenging for the virus to spread to individuals in a population (Desai & Majumder 2020).

The pandemic has had dire effects on South Africa, both socially and economically. Measures to minimise transmission were implemented across the globe and in South Africa (Stiegler & Bouchard 2020). These consisted of mandatory wearing of face coverings, hand hygiene, physical distancing, quarantining and lockdowns (Polack et al. 2020). The nationwide lockdown that shut down the economy resulted in widespread job losses, thereby exacerbating poverty which already plagues the nation. Actions that are taken to slow down the spread of COVID-19 and flatten the curve are only temporary measures. Research shows that herd immunity will need to be established as a long-term control measure. The aim in South Africa is to vaccinate more than 70% of the population (WHO 2022).

Vaccine hesitancy, however, is a barrier to achieving herd immunity and immunisation of most of the eligible population. In order to address vaccine hesitancy within a population, it is important to understand the root causes (Eskola et al. 2015).

The sample population of this study was those residing in Merebank, Wentworth and Bluff (Ward 68), KwaZulu-Natal. Participants in this area are exposed to high levels of pollution, given that it is an industrial hub. This has resulted in an increased incidence of respiratory diseases amongst members of the population (Xolo 2020). Since COVID-19 is a respiratory disease, it would be relevant to study the knowledge, attitudes and perceptions (KAP) of the population regarding the vaccine rollout to determine the level of vaccine acceptance in this community. This is also a low- to middle-income area that reflects the majority of the South African population, and therefore the findings of this study can be extrapolated to similar population groups in South Africa.

## Research methods and design

### Study design and setting

A quantitative cross-sectional study was conducted using an online self-administered KAP questionnaire available on Google Forms. This form could be completed on either a mobile device or a computer. Participants were given the option to complete the KAP questionnaire at the pharmacy or from a different location using the link provided. The study period was between April 2021 to September 2021.

This study was conducted at a retail pharmacy in Merebank, Wentworth and Bluff (Ward 68), which is located in the eThekwini Metropolitan Municipality in the KwaZulu-Natal province of South Africa. The pharmacy is located in a central shopping centre that is frequented by the community and services a vast majority of the residents, making it an ideal site to conduct the study.

### Study population and sampling strategy

The target population for the study included those residing in the geographical area of Merebank, Wentworth and Bluff (Ward 68). As per the 2011 census, that target population was 39 355 people (Wazimap 2021). This sample falls in a geographic location that is ridden with air pollution because of the many industries located around the residential area, and hence it was a prime location to conduct this study (Xolo 2020). The study participants were patients of the community pharmacy in the area. Participants have already developed a relationship with the pharmacy and felt more inclined to participate in the study for the progress and development of the community. Residents younger than 18 years old and health care workers were excluded from the study.

### Sample size determination

There are several formulas developed for sample size calculation that conform to different research situations (Cochran 2007), Accordingly, the sample size determination formula that was adopted for this study is:

$$n=\frac{\frac{Z^{2}PQ}{d^{2}}}{1+\frac{Z^{2}PQ}{Nd^{2}}}$$ [Eqn 1]

Where Z is the upper point of the standard normal distribution, which is 1.96, d is a clinically acceptable margin of errors, which is 5%, P is the expected prevalence of knowledge, which is 50%, *Q* = 1–*P* and the total number of targeted population is 39 355 (Wazimap 2021). The sample size was calculated to be 381. Taking into consideration the possibility of dropouts and unforeseen circumstances, a 10 percent nonresponse rate was added to the sample to make a maximum sample size of 419.

### Data collection

Data were collected using an online questionnaire divided into sections to collect data on the demographics and COVID-19 KAP of the population. The questionnaire took approximately 20 min – 30 min to complete and submit online via Google Forms. The study was piloted among 25 participants and was reworded for clarity and ambiguity prior to being rolled out.

### Data analysis

Data were entered into Microsoft Excel and was analysed using the Statistical Package for the Social Sciences (SPSS) version 26. Descriptive and inferential statistics were calculated, including the percentage and 95% confidence interval positive response to questions. Chi-square and Fisher’s exact test were used to test the association between KAP of the participants on the COVID-19 vaccine rollout versus sociodemographic variables. Binary logistic regression was used to determine the effects of sociodemographics on the KAP of the participants on the COVID-19 vaccine rollout. All the tests were two-sided, and all *p*-values reported were tested at α = 0.05 level. The results were then presented in the form of tables and charts.

### Ethical considerations

Ethical clearance to conduct this study was obtained from the Biomedical Research and Ethics Committee (BREC) at the University of KwaZulu-Natal (UKZN) (reference number: BREC00002569/2021). All participants were informed of the nature, aim and objectives of the study and signed an informed consent form.

## Results

### Demographics

A total of 459 individuals responded to the questionnaire; however, only 430 agreed to participate in the study and were included in the final analysis. Table 1 presents the background variables of the sample population. Participants aged 18–35 years old amounted to 44.4% of responses. A total of 59.8% of the respondents were female, with 36.4% being male. A majority of the participants were found to be Indian, who contributed a total of 62.6%. An estimated cumulative household income of greater than R15 000 was reported by 48.9% of the participants, followed by 19.5% having an income within the range R5001 – R10 000. Of the participants, 27.8% completed a matric, 26.9% completed an undergraduate degree and 18.5% completed a diploma. A majority of participants were employed prior to the pandemic (67.8%), of which 38.4% reported that their employment or income was severely affected.

**TABLE 1:** The sociodemographic characteristics of the survey participants (*n* = 430).

Background variables	Count	Percent (%)
**Age**		
18–25	114	26.5
26–35	77	17.9
36–45	71	16.5
46–55	92	21.4
56–65	47	10.9
> 65	29	6.7
**Gender**		
Female	256	59.8
Male	156	36.4
Nonbinary or transgender	8	1.9
Prefer not to say	8	1.9
**Race**		
Asian	10	2.3
Black	52	12.1
Mixed race	55	12.9
Indian	268	62.6
White	40	9.3
**Marital status**		
Divorced	33	7.7
Married	181	42.2
Single	184	42.9
Widowed	31	7.2
**The number of members in household**		
1–2	122	28.7
3–4	206	48.5
4–5	5	1.2
5–10	89	20.9
> 10	3	0.7
**How many children do you have?**		
None	194	45.2
1–2	172	40.1
3–4	54	12.6
5–6	5	1.2
> 6	4	0.9
**Estimated cumulative household income**		
R0 – R1000	16	3.9
R10 001 – R15 000	60	14.5
R1001 – R2500	16	3.9
R2501 – R5000	39	9.4
R5001 – R10 000	81	19.5
> R15 001	203	48.9
**What is your highest level of education?**		
Primary school	8	1.9
High school (Grade 8–11)	66	15.4
Matric	119	27.8
Postmatric certificate	41	9.6
Diploma	79	18.5
Degree	115	26.9
**Were you employed prior to the pandemic?**		
No	137	32.2
Yes	289	67.8
**How has the pandemic affected your employment or income?**		
Not at all	66	22.6
Mildly	43	14.7
Moderately	71	24.3
Severely	112	38.4

### Knowledge variables

#### COVID-19 knowledge

Table 2 presents the results of three COVID-19 knowledge questions. A total of 73.3% of the participants answered correctly in saying there is no cure for COVID-19, 86.3% of respondents correctly answered that it is possible for a person to be asymptomatic and still test positive and 86% of participants (*n* = 430) understood that it is possible to be reinfected with COVID-19.

**TABLE 2:** Responses to the three COVID-19 knowledge questions (*n* = 430).

COVID-19 knowledge items	Count	Percent (%)
**Is there a cure for COVID-19?**		
I do not know	80	18.6
No	315	73.3
Yes	35	8.1
**Is it possible for a person to be asymptomatic and still test positive for COVID-19?**		
I do not know	40	9.3
No	19	4.4
Yes	371	86.3
**Can you be reinfected with COVID-19?**		
I do not know	40	9.3
No	20	4.7
Yes	370	86.0

COVID-19, coronavirus disease 2019.

Table 3 summarises the overall knowledge of the participants (*n* = 430) based on several demographic variables. Of significance is the effect of the pandemic on employment (*p*-value: 0.001), news (*p*-value: 0.000/0.002), social media (*p*-value: 0.001/0.0014) and COVID-19 vaccine knowledge (*p*-value 0.000). The more severe the impact on income and employment, the greater the lack in overall knowledge of the participants. It was noted that 85.7% of participants who watched news had good overall knowledge, compared to those who did not watch news having only 57.1% with good overall knowledge. It can also be noted that the more frequently the participants watched the news, the better their overall knowledge. Participants with social media showed greater knowledge (84.1%) than those without social media (67.1%). The participants using social media for news updates proved to have better knowledge than those who did not use social media for news updates.

**TABLE 3:** Summary of background variables versus overall knowledge (*n* = 430).

Background variables	Overall knowledge				Chi-square *p*-value
	Not Good		Good		
	Count	%	Count	%	
**Age**					
18–25	22	19.3	92	80.7	0.141
26–35	12	15.6	65	84.4	
36–45	11	15.5	60	84.5	
46–55	16	17.4	76	82.6	
56–65	10	21.3	37	78.7	
> 65	11	37.9	18	62.1	
**Gender**					
Female	48	18.8	208	81.3	0.922
Male	31	19.9	125	80.1	
Nonbinary	1	12.5	7	87.5	
Prefer not to say	2	25.0	6	75.0	
**Marital status**					
Divorced	8	24.2	25	75.8	0.128
Married	26	14.4	155	85.6	
Single	39	21.2	145	78.8	
Widowed	9	29.0	22	71.0	
**The number of members in your household**					
1–2	26	21.3	96	78.7	0.880
3–4	36	17.5	170	82.5	
4–5	1	20.0	4	80.0	
5–10	18	20.2	71	79.8	
> 10	1	33.3	2	66.7	
**How many children do you have?**					
None	36	18.6	158	81.4	0.335
1–2	30	17.4	142	82.6	
3–4	12	22.2	42	77.8	
5–6	2	40.0	3	60.0	
> 6	2	50.0	2	50.0	
**Estimated cumulative household income**					
R0 – R1000	4	25.0	12	75.0	0.358
R10 001 – R15 000	11	18.3	49	81.7	
R1001 – R2500	3	18.8	13	81.3	
R2501 – R5000	10	25.6	29	74.4	
R5001 – R10 000	20	25.0	60	75.0	
> R15 001	29	14.3	174	85.7	
**What is your highest level of education?**					
Primary school	1	12.5	7	87.5	0.481
High school (Grade 8–11)	14	21.2	52	78.8	
Matric	26	21.8	93	78.2	
Postmatric certificate	10	24.4	31	75.6	
Diploma	15	19.0	64	81.0	
Degree	15	13.0	100	87.0	
**Were you employed prior to the pandemic?**					
No	31	22.6	106	77.4	0.223
Yes	51	17.6	238	82.4	
**How has the pandemic affected your employment or income?**					
Not at all	6	9.1	60	90.9	0.001*
Mildly	1	2.3	42	97.7	
Moderately	16	22.5	55	77.5	
Severely	28	25.0	84	75.0	
**Do you watch the news?**					
No	12	42.9	16	57.1	0.000*
Sometimes	21	31.3	46	68.7	
Yes	47	14.3	281	85.7	
**How often do you watch the news?**					
3–4 times a week	19	24.4	59	75.6	0.002*
Daily	36	14.8	208	85.2	
Monthly	1	5.6	17	94.4	
Never	12	42.9	16	57.1	
Weekly	11	20.4	43	79.6	
**Do you have any social media?**					
No	26	32.9	53	67.1	0.001*
Yes	55	15.9	291	84.1	
**Do you use your social media for news updates?**					
No	8	17.8	37	82.2	0.014*
Sometimes	18	26.9	49	73.1	
Yes	28	12.2	202	87.8	
**Do you share information regarding the pandemic without verifying if it is misinformation?**					
No	72	18.3	322	81.7	0.492
Not applicable	8	21.6	29	78.4
Sometimes	8	12.5	56	87.5
Yes	20	10.9	164	89.1

COVID-19, coronavirus disease 2019.

* , *p*-value < 0.05 is considered statistically significant, thus indicating that these background variables have a significant impact on overall knowledge.

#### COVID-19 vaccine attitudes

Table 4 presents the data collected on the participant’s vaccine attitudes. A total of 54.5% of the participants stated that because of the pandemic, they were more likely to receive the vaccine. Many of the participants (65%) stated that they would definitely take the COVID-19 vaccine, and 67.1% of participants stated that they would definitely encourage family and friends to take the vaccine. Just over half of the participants (51.5%) agreed that they would not take a vaccine that their general practitioner (GP) did not recommend. A total of 64.8% of participants agreed that a vaccine is crucial to end the pandemic, with 61.4% of the participants agreeing that the vaccine is extremely beneficial to their health.

**TABLE 4:** The participants’ (*n* = 430) attitudes towards COVID-19 and the COVID-19 vaccine rollout.

COVID-19 vaccine attitudes items	Count	Percent (%)
**How has the pandemic affected your views on vaccination?**		
I am less likely to opt to receive the vaccine.	42	9.9
I am more likely to opt to receive the vaccine.	235	55.2
My opinion on vaccination remains the same.	149	35.0
**Will you vaccinate against COVID-19?**		
I do not know.	53	12.4
I would probably get the vaccine.	49	11.5
I would probably not get the vaccine.	17	4.0
No, I will definitely not get the vaccine.	30	7.0
Yes, I will definitely take the vaccine.	277	65.0
**Will you encourage your family and friends to vaccinate?**		
I do not know.	63	14.7
I would probably encourage them to vaccinate.	38	8.9
I would probably not encourage them to vaccinate.	13	3.0
No, I will definitely not encourage them to vaccinate.	27	6.3
Yes, I will encourage them to vaccinate.	287	67.1
**I do not want a vaccine that my GP does not recommend.**		
Agree	102	24.1
Disagree	46	10.9
Neutral	124	29.3
Strongly agree	116	27.4
Strongly disagree	35	8.3
**A vaccine against COVID-19 is crucial to end the pandemic.**		
Agree	94	22.1
Disagree	22	5.2
Neutral	85	20.0
Strongly agree	182	42.7
Strongly disagree	43	10.1
**How beneficial is the COVID-19 vaccine to your health?**		
Extremely	255	61.4
Moderately	92	22.2
Not at all	38	9.2
Slightly	30	7.2

GP, general practitioner; COVID-19, coronavirus disease 2019.

**FIGURE 1:**
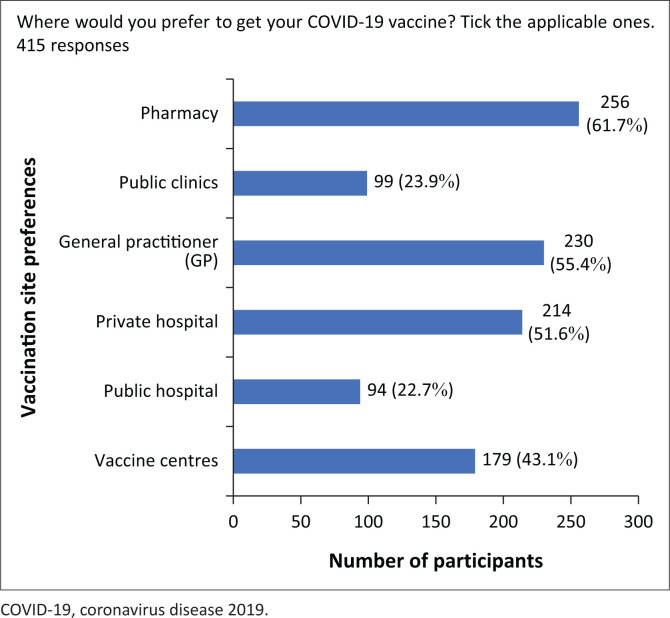
Bar graph showing the vaccination sites participants preferred.

Figure 1 shows the vaccination sites at which the participants (*n* = 430) preferred to receive their COVID-19 vaccine. Pharmacies were selected as the preferred vaccination site (61.7%), followed by GP (55.4%) and private hospital (51.6%). Public clinics and public hospitals were least preferred at 23.9% and 22.7%, respectively.

#### COVID-19 perceptions

Table 5 presents data on the participants’ (*n* = 430) overall perceptions of COVID-19. Most participants (94.3%) agreed that COVID-19 is a major problem in South Africa. When considering vaccine safety and access, only 50.8% of the participants thought the COVID-19 vaccine was safe, and 53.7% believed it would be easy to obtain the vaccine. A total of 66.3% of the participants said they were not hesitant to take the vaccine, whilst 51.5% acknowledged that their personal experiences surrounding the pandemic would affect their decision of taking the vaccine.

**TABLE 5:** The perceptions of the participants (*n* = 430) towards the COVID-19 vaccine rollout.

COVID-19 vaccine perceptions items	Count	Percent (%)
**How much of a problem is COVID-19 in South Africa? *n* = 420**		
Not a problem	2	0.5
Insignificant problem	1	0.2
Slight problem	21	5.0
Major problem	396	94.3
**Do you think the COVID-19 vaccine is safe? *n* = 425**		
Maybe	163	38.4
No	46	10.8
Yes	216	50.8
**Do you feel it will be easy to obtain the vaccine once it becomes available? *n* = 423**		
Maybe	129	30.5
No	67	15.8
Yes	227	53.7
**Will your personal experience of the COVID-19 pandemic affect your decision of taking the vaccine? *n* = 423**		
Maybe	40	9.5
No	165	39.0
Yes	218	51.5
**Are you hesitant to take the COVID-19 vaccine? *n* = 421**		
No	279	66.3
Yes	142	33.7

COVID-19, coronavirus disease 2019.

**FIGURE 2:**
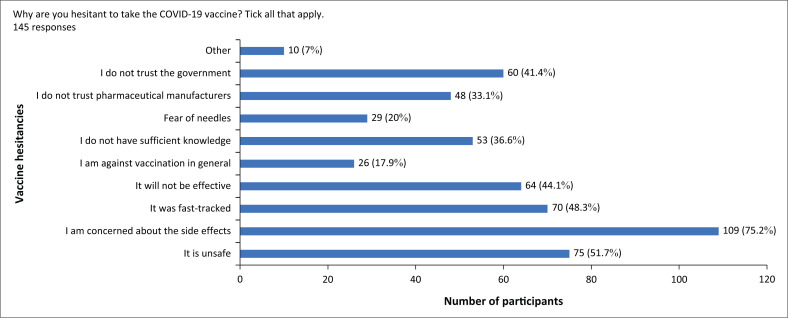
The bar graph shows reasons for participants being hesitant to take the COVID-19 vaccine.

Figure 2 presents the descriptive statistics for reasons behind vaccine hesitancies. The reasons reported were concerns surrounding side effects of the vaccines (75.2%), vaccine safety (51.7%), fast-tracking of vaccine development (48.3%), vaccine efficacy (44.1%) and government mistrust (41.4%).

#### News and social media

The following responses were gathered from questions surrounding news and social media. The study found that many participants (77.5%) watch the news, with 57.8% watching the news daily. A majority of participants (81.4%) have social media platforms, and 67.3% use social media for their news updates. A total of 42.7% of participants stated that they mostly believe what they read online. The most popular sources of information were televised news (78.5%), social media (62%), online newspapers (58.1%) and radio news (53.5%).

## Discussion

### Demographics

According to the 2011 census, about 29% of the population in Merebank, Wentworth and Bluff (Ward 68) fall between the ages of 20–39 years old (Wazimap 2021). The study noted 44.4% of the respondents were aged 18–35 years old. The large number of responses in this age group could be accounted for by the fact that they are more likely to have access to online platforms to complete the questionnaire. Most participants of the study were female and were of Indian (62.6%) race. A study conducted in Canada compared the health-seeking behaviours of men and women to physical and mental health concerns and found that women are more likely to visit their primary health care providers than men on health-related concerns (Thompson et al. 2016).

### COVID-19 knowledge

Knowledge in terms of healthcare represents facts, experiences and capabilities developed through education and/or experience of a subject related to health care (Chin et al. 2011). Through gaining knowledge, patients are equipped with reliable, evidence-based information that ensures they make sound decisions regarding their health care. More so now with the COVID-19 pandemic, individuals need to have adequate knowledge regarding COVID-19 and the available vaccines to improve vaccine acceptance amongst the population.

The three COVID-19 knowledge questions asked if there is a cure for COVID-19, if it is possible to be asymptomatic and still test positive for COVID-19 and if reinfection is possible. Overall, the mean COVID-19 knowledge in the population based on these three questions was 81.86%, and therefore it can be concluded that the population has a good level of knowledge and understanding. Knowledge is an essential predictor of health behaviour, and therefore attention needs to be given to those participants who had low knowledge levels (Lee, Kang & You 2021). The reason for this is the effect knowledge will have on an individual’s attitudes and perceptions surrounding COVID-19 vaccination, thereby ultimately impacting the vaccine rollout.

At present, there is no cure for COVID-19; however, the COVID-19 vaccine has been proven to reduce the severity of the disease and prevent life-threatening illness should one contract the virus (WHO 2021a). It is important that the population understand that the vaccine is not a cure but rather a preventive measure that protects against severe disease. By making the population aware of this, it will help enforce the continued use of a face covering, practising physical distancing and maintaining hand hygiene after vaccination (WHO 2021b).

Asymptomatic infections have been found to have the same infectivity as symptomatic infections and therefore play a significant role in COVID-19 transmission prevention and control (Gao et al. 2021). The population needs to be aware of this and ensure isolation and screening if exposed to the virus, regardless of whether they present with symptoms. This will assist in bringing down the infectivity rate in the country (Gao et al. 2021).

Knowledge was significantly associated with employment or income, watching the news and frequency of watching the news, having social media and using social media for news updates.

It was noted that participants whose employment was affected severely had lower knowledge than those whose employment was affected mildly or not at all. The Africa Centre for Disease Control and Prevention survey found that participants who were employed believed the COVID-19 vaccine was safe in comparison to those who were unemployed (Ask Afrika 2021). This then shows an increased level of understanding and trust in the vaccine with those who are employed. The increased knowledge can be attributed to discussions surrounding COVID-19 vaccination by employers and colleagues. Employers can assist with improving vaccine uptake by providing employees with COVID-19 information through work-based education programmes. Furthermore, policies and practices should be created to provide support to the employees. On-site vaccination programmes can be implemented where possible, either through the occupational health clinics or via mobile vaccination facilities (Workplace Vaccination Program 2021).

Participants watching the news had a significantly higher knowledge than those who watched the news sometimes or not at all. Additionally, knowledge significantly improved with the frequency of watching the news; that is, respondents watching the news daily had better knowledge than those watching the news 3–4 times a week or weekly. Social media plays an important role today as a tool not only to connect with others but also to stay abreast on current affairs (The Lancet Infectious Diseases 2020). Most of the participants who had social media used it for news updates and subsequently had better knowledge.

Vaccine hesitancy is influenced by a variety of factors; however, knowledge and awareness play an integral role in vaccine acceptance (Yaqub et al. 2014). A study carried out in South Korea researched the association between KAP towards COVID-19. The results showed knowledge directly affects the attitudes and behaviours of participants, particularly with the implementation of preventive strategies (Lee et al. 2021). Individuals with better vaccine knowledge will understand its importance and benefits. This will likely increase vaccine acceptance through fostering positive attitudes and beliefs (Zheng, Jiang & Wu 2021). A study conducted on general vaccine knowledge across six vaccines found that knowledge has a positive effect on vaccine behaviour and intention (Schulz, Hartung & Schulz 2021).

The key to building confidence in the COVID-19 vaccine lies with identifying knowledge gaps and developing strategies to empower patients with factual and reliable data to make informed decisions regarding vaccination and immunisation.

### COVID-19 vaccine attitudes

Attitudes comprise a favourable or unfavourable assessment of a concept. Health attitudes, then, are an extensive assessment of one’s own health and health needs (Chipperfield, Bailis & Perry 2021). Typically, a positive health attitude reflects a positive overall health status and is associated with desirable health-related consequences (Chipperfield et al. 2021).

In this survey on community-based attitudes towards the rollout of the COVID-19 vaccine, 65% of the participants said they would definitely vaccinate, and 67.1% said they would encourage family and friends to vaccinate. The latest Independent Polling System for Society (IPSOS) survey shows that the United Kingdom shows the highest level of intent to vaccinate compared to the 15 countries surveyed. The IPSOS study conducted in January 2021 showed a 61% intent to vaccinate amongst South African participants (Boyon 2021). The University of Johannesburg’s Centre for Social Change and Human Sciences Research Council’s (HSRC) democracy survey found that 67% of participants intended to vaccinate (Runciman et al. 2021). The aim in South Africa is to vaccinate more than 70% of the population (WHO 2022). This is going to be a difficult task, given that this study found only 65% of participants are willing to vaccinate. To improve vaccine acceptance and create positive attitudes on vaccination, the government and health care workers need to address the reasons for vaccine hesitancy and provide education to the population.

A total of 64.8% of participants agreed that a vaccine is crucial to end the pandemic, with 61.4% believing that the COVID-19 vaccine was extremely beneficial to their health. It is a social responsibility of participants to get vaccinated, thereby protecting themselves and others (WHO 2021a).

Doctors and pharmacists play a vital role in the COVID-19 vaccination rollout, and the government should leverage their influence on the community to promote and encourage a speedy uptake of the COVID-19 vaccine. There are several vaccination sites available across the country. Of all the sites available to participants, the site that received 61.7% preference was a pharmacy, followed by a 55.4% preference for a GP and then a 51.6% preference for a private hospital. The Council for Medical Schemes COVID-19 survey found that GPs and pharmacists were the preferred vaccination sites (Willie & Skosana 2021).

Community pharmacists have established a trustworthy relationship with their patients and can therefore drive effective immunisation programmes, thereby improving their community’s vaccine uptake and acceptance (Ballard et al. 2021). Pharmacists are often the most accessible health care workers, especially amidst the pandemic (Elbeddini et al. 2020). The successful implementation of National Health Insurance (NHI) in a low- to middle-income country like South Africa to ensure universal health coverage will require collaboration between all health care professionals. The role of pharmacists need to be reassessed to expand their skillset to focus on primary health care (Naidoo et al. 2020). Community pharmacists are often the first point of care for most patients. Their knowledge, accessibility and extended trading hours make pharmacists an ideal for enhancing primary health care coverage under the NHI (Bheekie & Bradley 2016). As such, it is imperative that they clarify misconceptions surrounding the COVID-19 vaccine and deliver factual and reliable information to their patients (Elbeddini et al. 2020).

Half of the participants agreed that they did not want a vaccine that their GP did not recommend. A hospital-based study in Sydney, Australia showed that a physician recommendation was a key influence on a patient’s attitudes towards vaccination, as majority of patients in the study did not a vaccine that was not recommended by their GP (Ridda et al. 2008). This shows the influence health care providers may have on vaccine hesitancy amongst the general population, and it is of utmost importance that health care providers relay a positive message. Vaccines need to be recommended to all eligible patients by health care workers whenever possible (Ridda et al. 2008).

Although only 65% of participants agreed to vaccinate, increasing vaccination acceptance is attainable through promoting positive vaccine attitudes amongst the population. Reaching the target population for herd immunity is well within reach, and South Africa’s vaccination rollout is advancing in a promising manner.

### COVID-19 vaccine perceptions

One of the determinants of COVID-19 vaccine acceptance is public perceptions. This study focused on the perceived risk of COVID-19 in South Africa, perceptions on vaccine safety and vaccine hesitancies.

The study found that 94.3% of participants perceived COVID-19 as a major problem, yet vaccine uptake is significantly lower. This could be attributed to perceptions on vaccine safety, seeing as only 50.8% considered the vaccine safe.

When assessing vaccine hesitancy, 33.3% of participants were hesitant to take the vaccine for various reasons. The most common reasons included concerns about the side effects (75.2%), vaccine safety (51.7%), the fact that it was fast-tracked (48.3%), vaccine effectiveness (44.1%) and not trusting the government (41.4%) and pharmaceutical manufacturers (33.1%). According to three other studies conducted in South Africa, the most common reason reported for vaccine hesitancy was concerns about the side effects of and the efficacy of the vaccine (Cooper, Van Rooyen & Wiysonge 2021). Similarly, an international survey conducted in low- and middle-income countries (LMICs) on factors affecting vaccine acceptance found that the main reasons for vaccine hesitancy were the potential side effects and reduced confidence in vaccine effectiveness (Bono et al. 2021).

### News and social media

At present, many across the world are trying to fight the COVID-19 pandemic; however, this fight has only been fuelled by the ‘infodemic’ caused by the spread of false news, misinformation and conspiracy theories through social media platforms and certain news outlets. This then creates mistrust in governments, health care providers and health care institutions (The Lancet Infectious Diseases 2020).

Information on the pandemic is rapidly changing as new evidence and research becomes available. News outlets and the government are constantly reporting information as it becomes available, some of which may contradict previous information shared with the public. Governments then retract their recommendations based on new emerging data and in doing so create doubt and uncertainty in some citizens (The Lancet Infectious Diseases 2020). For example, South Africa planned on rolling out the Oxford-AstraZeneca vaccine; however, a study found that it did not protect against the 501.V2 variant, and so the rollout was halted (Mahase 2021). Stopping the vaccine rollout negatively impacted the population’s confidence and trust in the vaccine (Ask Afrika 2021).

Through the COVID-19 pandemic, social media communication has acted as an ally because of the speed at which information can be shared across the globe, especially between the scientific community and health care workers. However, despite this, social media has also caused chaos and mayhem because of the rampant spread of misinformation across all platforms (Venegas-Vera, Colbert & Lerma 2020).

As much as social media has a role in the spread of misinformation, it is through these same platforms that the population is provided up-to-date information during a pandemic at an unprecedented rate. It is therefore important to educate the population on the need to debunk false information and to stop the spread of information from unverified sources (Obi-Ani, Anikwenze & Isiani 2020).

### Limitations of the study

South Africa was in its second wave of the COVID-19 pandemic when this study was conducted; and for that reason, many patients were not coming into the pharmacy but opted for home deliveries instead. This reduced the number of patient participants available in the pharmacy to take the study.

A standard operating procedure at the pharmacy was to minimise contact with shared surfaces, and so the survey had to be conducted via an online platform. Seeing as this was a low- to middle-income area, all participants may not have access to electronic devices.

As SARS-CoV-2 is a novel virus, information regarding COVID-19 and vaccination is very limited and constantly changing as new information becomes available.

To encourage participation, the study did not collect any personal information, including e-mail addresses, hence participants were unable to receive copies of their responses or results of the study.

### Strengths of the study

The study was conducted in a low- to middle-income area, and therefore the results of this study can be used as an indication for similar populations in South Africa.

The study managed to identify reasons for vaccine hesitancy, and this data can be used in addressing these areas of concerns, thereby promoting vaccine acceptance.

The maximum required sample size for this study was found to be 419; however, a total of 430 participants had participated in the study, thereby surpassing this requirement.

## Conclusion

The study set out to determine the KAP of a patient population in Merebank, Wentworth and Bluff (Ward 68), South Africa, on the COVID-19 vaccine rollout. An online quantitative questionnaire was provided to patients of Natraj Care Pharmacy. The responses were collected on Google Forms, and the results were analysed. The study found that about two-thirds of the population intended to receive the COVID-19 vaccine. In addition, reasons for vaccine hesitancies were assessed, and the two major concerns that need to be addressed were the potential side effects and the vaccine efficacy. Furthermore, news and social media were associated with knowledge and play a vital role in dissemination of COVID-19 vaccine information. Having said that, it also contributes to the current infodemic, whereby misinformation and conspiracy theories are spread at an alarming speed. Education campaigns need to be customised to not only provide the population with reliable and vetted vaccine information but also to address specific concerns or hesitancies present. To reach herd immunity and ensure the prevention of increased morbidity rates, there needs to be a rise in vaccine acceptance across South Africa and the world.
